# *Actinobacillus pleuropneumoniae* serovar 8 predominates in England and Wales

**DOI:** 10.1136/vr.103820

**Published:** 2016-08-16

**Authors:** Y. Li, J. T. Bossé, S. M. Williamson, D. J. Maskell, A. W. Tucker, B. W. Wren, A. N. Rycroft, P. R. Langford

**Affiliations:** 1 Section of Paediatrics, Imperial College London, St Mary's Campus, London W2 1PG, UK; 2 APHA—Bury St Edmunds, Rougham Hill, Bury St Edmunds, Suffolk IP33 2RX, UK; 3 Department of Veterinary Medicine, University of Cambridge, Madingley Road, Cambridge CB3 0ES, UK; 4 Faculty of Infectious & Tropical Diseases, London School of Hygiene & Tropical Medicine, Keppel Street, London WC1E 7HT, UK; 5 Department of Pathology and Pathogen Biology, Royal Veterinary College, Hawkshead Lane, North Mymms, Hertfordshire AL9 7TA, UK

**Keywords:** Actinobacillus pleuropneumoniae, Serotyping, Bacterial diseases

*Actinobacillus pleuropneumoniae* is a major cause of pleuropneumonia, an acute or chronic lung disease of pigs that causes significant morbidity, mortality and economic losses in the worldwide pig industry (Bossé and others 2002, Gottschalk 2012). There are 15 established serovars of the bacterium determined by the composition of the capsular polysaccharide (Gottschalk 2012) with another proposed based on serology alone (Sárközi and others 2015). Determining the serovar allows insights into the epidemiology of *A. pleuropneumoniae*, for example, to map outbreaks of disease, to identify introductions of serovars not previously detected in countries and to assist in the formulation of bacterin vaccines. Serovar prevalence varies from country to country and with time (Dubreuil and others 2000, Gottschalk 2015). For example, currently serovars 5 and 7 dominate in Canada (Gottschalk and Lacouture 2015), whereas until the 1990s, it was serovar 1 (Gottschalk 2015). In Australia, serovar 15 is highly prevalent (Turni and others 2014) but is rarely reported elsewhere, although it has been recently found in Canada (Gottschalk and Lacouture 2014) and possibly also in Japan (Koyama and others 2007).

The last prevalence study in the UK analysed *A. pleuropneumoniae* clinical isolates collected in England and Wales between the years 1995 and 2007, with serovar 8 predominant (O'Neill and others 2010). In that study, serovar was assigned on the basis of PCR amplification of chromosomal capsule-specific regions; immunological serotyping by slide agglutination having been shown erroneously to overestimate serovars 3 and 6 and underestimate serovar 8 prevalence. Similar underestimation and overestimation were also found in a study of Canadian isolates (Gottschalk 2015). In this study, the authors sought to determine whether the serotype prevalence of *A. pleuropneumoniae* in England and Wales had changed since 2007 and, in particular, whether new serovars, such as 15, were now present. The isolates evaluated were obtained from clinical cases of diseases in pigs due to *A. pleuropneumoniae* submitted to the Animal Health and Veterinary Laboratories Agency—Weybridge (AHVLA), now the Animal and Plant Health Agency (APHA), or Royal Veterinary College Diagnostic Laboratories. Isolates were collected between 2008 and 2014. For each year, the number of isolates (in brackets) is as follows: 2008 (N=14); 2009 (N=17); 2010 (N=8); 2011 (N=10); 2012 (N=30); 2013 (N=19) and 2014 (N=15). Bacteria were grown on plates comprising Brain Heart Infusion Agar supplemented with 10 µg/ml nicotinamide adenine dinucleotide. Initially, serotyping was assessed by a multiplex PCR that can distinguish between serovars 1–3, 5–8, 10 and 12 (Bossé and others 2014). The PCR was based on the authors’ original serovar 3–6–8 PCR (Zhou and others 2008) but extended to cover all of the serovars reported in England and Wales in the authors’ 2010 study (O'Neill and others 2010) and those reported to be prevalent in Europe and North America (Dubreuil and others 2000). A serovar 15-specific PCR (Turni and others 2014) was subsequently used on strains that were untypable by the 1–3, 5–8, 10 and 12 multiplex PCR. Genomic DNA purified using the QIAamp DNA Mini Kit (Qiagen) or single colonies were used as the source of DNA template for PCR reactions as previously described (Bossé and others 2014). In addition to serovar-specific amplicons, the multiplex PCR amplifies a region of the *A. pleuropneumoniae*-specific *apxIVA* gene (Schaller and others 2001, Bossé and others 2014). All isolates investigated in this study produced an *apxIVA* amplicon confirming that they were *A. pleuropneumoniae*. Comparison of the seroprevalence of *A. pleuropneumoniae* in England and Wales in this and the authors’ previous study is shown in Table 1.

**TABLE 1:** Percentage serovar distribution of *A. pleuropneumoniae* isolates in England and Wales-based studies

Percentage of isolates (number of isolates) in range of years indicated		
Serovar	1995–2007*	2008–2014†
2	3.4 (13)	6.2 (7)
3	0.5 (2)	–
6	10.1 (38)	7.1 (8)
7	5.3 (20)	8.0 (9)
8	78.0 (295)	71.7 (81)
12	2.7 (10)	3.5 (4)
NT	–	3.5 (4)

*O'Neill and others (2010)

†Present study

NT, non-typable

As in the authors’ 2010 study, serovar 8 was the most predominant in years 2008–2014 in England and Wales, with a comparatively smaller percentage of serovars 2, 6, 7 and 12. The England and Wales serovar profile contrasts with that found in other European countries. For example, serovar 9/11 predominates in the Czech Republic (Kucerova and others 2005) and serovar 2 in Denmark (Klausen and others 2007), Norway and Sweden (Gottschalk 2012). No serovar 3 isolates were found, possibly reflecting the known low virulence of this serotype (Rosendal and others 1985), and that the *A. pleuropneumoniae* isolates evaluated were from clinically confirmed cases. Four isolates were non-typable (NT) and subsequent PCR testing established that these were not serovar 15 (Turni and others 2014). Due to their low numbers, the NT isolates were not tested further. The NT isolates could be serovars 4, 9/11, 13, 14, the newly described serovar 16 or variants such that the primers used did not anneal to genomic DNA. Among these, only serovar 9 has been previously reported in Great Britain (McDowell and Ball 1994) or Scotland (Anon 2013).

In summary, the authors’ results suggest that serovar 8 *A. pleuropneumoniae* remains the predominant serovar causing clinical disease outbreaks in England and Wales, as in the authors’ previous study. Any vaccine to be used in the UK to prevent disease caused by *A. pleuropneumoniae* should have a component targeting serovar 8 isolates and, for near-maximal coverage, additionally serovars 2, 6, 7 and 12.
